# Domestic Correspondence

**Published:** 1884-07

**Authors:** 


### DOMESTIC CORRESPONDENCE.

LETTER FROM BOSTON. *Death of Professor Ellis—Resignations at the Massachusetts General Hospital—Boston's New Sewerage System—Annual Meeting of the Massachusetts Medical Society—Posterior Plaster Splint—The Admission of Women.*

BOSTON, June 24, 1884.

You know that medical men are very fond of professional gossip, and had you lived in Boston for the last few months your appetite for medical small talk would have been satiated. What with changes in the Medical School and resignations at the Massachusetts General Hospital, we have been indeed amused. The first change was the irreparable loss by death to the Medical School, of Professor Calvin Ellis, who had for many years filled the Jackson chair of clinical medicine. Dr. Ellis' memory has been honored by a stated meeting of the District Society, and the encomiums there offered were eloquent and justly deserved. A man of simple habits, upright conduct in all the paths of life, a careful instructor, a trusted family counselor, and a highly valued consultant. He left a competency, the greater part of which goes to the Harvard Medical School. A worthy successor has been selected in the person of Dr. Robert T. Edes, the Professor of Therapeutics, who has been transferred to the chair of clinical medicine.

An unpleasant state of affairs has arisen at the Massachusetts General Hospital, where the two senior surgeons, Drs. Bigelow and Hodges, have tendered their resignations to the Board of

Trustees, owing, it is said, to the refusal of the Board to appoint as house pupil the candidate nominated by the majority of the staff. For years a feeling has existed that appointments at that institution in many cases were the result of favoritism, and the acceptance by the Board, of a minority report has been the "casus belli." It is thought that the gentlemen will remain, as the Trustees have already asked them to withdraw their resignations. Their loss to any hospital would be a great one, but especially is it to be regretted that men occupying the position they do in the profession should become involved in an almost personal quarrel, and feel it incumbent to resign in a huff. In justice to the gentlemen, let me say that it is generally conceded that they were right in the issue taken, but the principle of tendering resignations where one cannot have one's own way is wrong; and we retrospect upon our juvenile days when a cross in our play led us to exclaim, "I won't play!"

Our new system of sewerage which has been put in operation this year is working very satisfactorily; the city now has a system of sewerage second to none in the world, and which has been constructed at an expense of between five and six millions of dollars. The work was begun seven years ago, with the object of intercepting the sewage of the city as it flows in the existing sewers and conveying it to a point where it may be discharged with safety to the public health. The intercepting sewers follow the marginal streets of the city as far as practicable, that the sewage may be intercepted near the outlets of the old sewers. The new system allows the sewage to flow at all times, regardless of the condition of tide or weather. The entire sewage of the city is collected in a large reservoir covering several acres at Moon Island. This reservoir will be emptied twice in twenty-four hours, it being so situated that the ebb tidal current will sweep the sewage far out to sea.

The system was designed by a commission appointed by the city government in 1875, consisting of Mr. E. S. Chesborough, City Engineer of Chicago; Mr. Moses Lane, City Engineer of Milwaukee, and Dr. C. F. Folsom, Secretary of the Massachusetts State Board of Health.

The one hundred and third meeting of the Massachusetts Medical Society was held in Boston on June 10th and 11th. The morning of the 10th was spent in visits by the members to the various hospitals. At noon the society assembled at the new Harvard Medical School to witness experiments and a demonstration of "Modern Methods of Research in Physiology," by Professor H. P. Bowditch. The laboratories were shown, where experiments were in progress under the direction of Professor Bowditch's assistants. Following these demonstrations, Dr. Thomas Dwight, the new Professor of Anatomy, who has been very successful in his teaching the past season, then addressed the society on "Modern Methods in Anatomy," showing superb frozen sections and anatomical preparations prepared by corrosion and by the use of Wood's fusible metal.

In the afternoon Dr. George W. Gay gave a demonstration of the application of the plaster posterior splint in the treatment of fractures of the leg. This method is largely used in Boston, and I can heartily commend it. The advantages of the splint are, its applicability to all fractures of the leg. Possibly certain Pott's fractures, where there is marked lateral displacement, should be excepted. It is an adaptable shell of plaster of Paris which is made for each individual fracture. It can always be readily opened—in fact, is always open—so that the limb can be under constant supervision; hence there may be no fear from sloughing or gangrene, the result of unrelieved pressure. It holds the fragments firmly in position, and the fracture remains "put." It can be easily removed and reapplied without impairing its integrity. The fractured limb having been wrapped in sheet wadding, a piece of crinoline long enough to extend from the toes to above the knee and wide enough to encircle the limb, is laid beneath the limb and closely wrapped around it, with the exception of a space an inch wide on its anterior aspect. This serves as a pattern from which to cut eight or ten similar pieces. These are slashed from their edge inward, especially at the heel, to allow their adaptation to the limb. The outer layer of gauze, which is larger than the rest, is folded over on the inner layer, thus binding the several layers of gauze and plaster together into a common whole. A plaster paste is then made, which is interlaid between the various pieces of foundation gauze. The splint is now adjusted to the limb, a roller bandage is applied over this, and the splint is complete. In a few hours the outer bandage may be removed, the wadding cut up and buckled straps applied to keep up the desired compression.

At the conclusion of this paper the society was called to order by President Hosmer. After a rather stormy discussion and a lamentable display of ignorance of parliamentary law by both President and Fellows, by-law I. was amended to read as follows: "Candidates for admission into the Massachusetts Medical Society may be either *male* or *female*, and every candidate must by proper credentials and examinations satisfy the censors of said society that he possesses the following qualifications, etc." Thus the question of the admission of women to the Massachusetts Medical Society is settled. At the meeting of the society three hundred and thirty-two members out of fifteen hundred and twenty-nine were present, of whom two hundred and nine voted to admit women and one hundred and twenty-three against their admission. At the Councilors' meeting, of one hundred and ten present out of one hundred and seventy-two Councilors sixty-three voted in favor of admission of women and forty-seven against it. The action of the society places it on a more favorable footing with the laity, whatever the judiciousness of the movement may be. Accepting that a certain number of well-educated women will practice medicine, that the State in chartering the Massachusetts Medical Society intended it to act as a licensing body, and that a widespread public opinion demanded their admission, the Censors of the society could not long have refused to consider a female's qualifications for admission to the Massachusetts Medical Society. The annual dinner passed off with unusual brightness under Dr. George B. Shattuck, who acted as anniversary chairman. Dr. Charles D. Homans, the President-elect, suggested that the present scene of the anniversary banquet might, with the advent of women into our ranks, be changed in twenty or thirty years to a ball-room. Let me say, however, that the general impression is that there will be hardly a "baker's dozen" who desire to enter the Massachusetts Medical Society.

